# Increased risk of rheumatoid arthritis among patients with Mycoplasma pneumonia: A nationwide population-based cohort study in Taiwan

**DOI:** 10.1371/journal.pone.0210750

**Published:** 2019-01-14

**Authors:** Kuo-An Chu, Weishan Chen, Chung Y. Hsu, Yao-Min Hung, James Cheng-Chung Wei

**Affiliations:** 1 Department of Internal Medicine, Division of Chest Medicine, Kaohsiung Veterans General Hospital, Kaohsiung, Taiwan; 2 Department of Nursing, Shu-Zen Junior College of Medicine and Management, Kaohsiung, Taiwan; 3 School of Medicine, National Yang Ming University, Taipei, Taiwan; 4 Management Office for Health Data, China Medical University Hospital, Taichung, Taiwan; 5 College of Medicine, China Medical University, Taichung, Taiwan; 6 Graduate Institute of Clinical Medical Science, China Medical University, Taichung, Taiwan; 7 Department of Emergency Medicine, Kaohsiung Veterans General Hospital, Kaohsiung, Taiwan; 8 Yuhing Junior College of Health Care and Management, Kaohsiung, Taiwan; 9 Division of Allergy, Immunology and Rheumatology, Chung Shan Medical University Hospital, Taichung, Taiwan; 10 Institute of Medicine, Chung Shan Medical University, Taichung, Taiwan; 11 Graduate Institute of Integrated Medicine, China Medical University, Taichung, Taiwan; Universiti Sains Malaysia, MALAYSIA

## Abstract

**Objective:**

An association between Mycoplasma pneumonia (MP) and rheumatoid arthritis (RA) had been reported in animal studies for decades. However, clinical evidence for this association is lacking. Therefore, this study aimed to provide epidemiologic evidence to clarify the relationship between MP and development of RA.

**Methods:**

This 13-year nationwide, population-based, retrospective cohort study analyzed the risk of RA in a cohort of MP patients. We cross linked and compared the database of those with catastrophic illnesses to make sure the diagnoses of RA are correctly labeled. We selected 116,053 hospitalized patients diagnosed with MP between 2000 and 2012 from the Taiwan National Health Insurance Research Database and 464,212 matched controls at a 1:4 ratio by age, gender, and index year, in relation to the risk of developing RA. The follow-up period referred to the initial diagnosis of MP until the date of RA diagnosis, censoring of RA, or 31^st^ December 2013. The Cox proportional hazard model was used to analyze the association between MP and incidence of RA among patients with different potential risks.

**Results:**

The adjusted hazard ratio (HR) for incidental RA in the MP group was 1.37 (95% confidence interval CI = 0.87–2.16), compared to non-MP controls. Stratified analysis revealed that the adjusted HR was 3.05 (95% CI = 1.16–7.99, p = 0.02) in a subgroup of patients over the age of 65.The adjusted HR of RA for the MP group among aged ≦19 years and ≥ 65 years was 3.19 (95% CI = 1.04.9.76) and 4.14 (95% CI = 1.27,13.4) within the first 2 years of follow-up.

**Conclusion:**

This cohort study demonstrated that patients with MP had a higher risk of developing RA, especially in the first 2 years, in those aged younger than 19 and over 65.

## Introduction

Autoimmune diseases have been reported to be globally on the rise and are now a public health concern as threatening as cancer and heart disease [1, 2]. Nevertheless, little is known regarding what environmental factors have triggered the increase in incidence of autoimmune diseases, other than genetic factors [2]. Of the known environmental factors, infections are thought to play a major role in stimulating the development of autoimmune diseases, which are often caused by particular pathogens [3–6] in viruses, bacteria and protozoa through molecular mimicry, epitope spreading, or bystander activation [7, 8]. Other pathogens, on the other hand, are found to help avert immune dysregulation, and protozoan infections may even prevent autoimmune disorders [8, 9].

As one of the most common inflammatory autoimmune diseases, rheumatoid arthritis (RA) has a global pervasiveness ranging from 0.4% to 1.3%[10–12], with an age-adjusted annual incidence rate 15.8 per 100,000 in Taiwan [13, 14]. The etiology of RA may result from an interaction between genetic factors and environmental exposure, as demonstrated in previous studies performed using animal models which shared homogeneous genetic and environmental backgrounds with humans. For instance, mycoplasma infections have been shown to induce experimental arthritis in mice or rats [15, 16]. However, the treatment of experimental animals with microbes occurs differently to infection of the human body with microbes. Though the arthritis symptoms found in experimental animals resembled joint inflammation observed in human RA, they did follow the natural course of RA seen in clinical RA patients [10].

*Mycoplasma pneumoniae* is one of the most common causes of atypical pneumonia in the United States and other parts of the world [17, 18]. The microbes are generally present in respiratory tract infections but may cause pneumonia alongside many extrapulmonary manifestations. Rheumatologic symptoms like polyarthralgia and myalgia are believed to result from immune-mediated mechanisms [19]. A number of small-scale clinical studies have suggested a possible role for mycoplasma in the synovium of RA patients [20–23]. Therefore, further human epidemiological studies are called for to elucidate the association of RA and Mycoplasma. As an endemic area of Mycoplasma pneumonia (MP), Taiwan has MP as the second commonest causative pathogen for community-acquired pneumonia among the adults [24]. Consequently, a lack of epidemiological studies of autoimmune diseases such as RA may hinder the country in developing adequate control of prevalent infections including MP. The longitudinal cohort study was conducted in order to explore the epidemiological relationship between MP and the subsequent development of RA.

## Materials and methods

### Data source

The data used in this study was obtained from the Taiwan National Health Insurance Research database (NHIRD) and was provided by the National Research Institutes for research purposes. The Taiwan National Health Insurance (NHI) program was established in 1995, and currently covers over 99% of the population in Taiwan.

Data in the NHIRD includes inpatient admissions records including billing of all beneficiaries enrolled in the NHI program. We cross linked and compared the database of those with catastrophic illnesses to make sure the diagnoses of RA are correctly labeled. Patients with RA are issued with a catastrophic illness certificate and are exempted from co-payments. For the patients’ privacy, their identities were encrypted before being released by the National Research Institutes. This study was approved by the Institutional Review Board of and the Hospital Research Ethics Committee of China Medical University (Institutional Review Board permit number: CMUH-104-REC2-115).

### Study subjects

The subjects in this study were hospitalized patients infected with MP (International Classification of Diseases, Ninth Revision, Clinical Modification [ICD-9-CM] codes 483.0) from 2000 to 2012. The index date was defined as the date of a diagnosis of MP. Patients with a previous diagnosis of RA (ICD-9-CM code 714) were excluded. Those who withdrew from the NHI program before the index date were also excluded. The selected comparison group was the inpatients who had never been diagnosed with MP. The ratio of the comparison group to the MP group was 1:4 matched by age, gender, and index year.

### Outcome and relevant variables

The main outcome was the diagnosis of RA, classified as a catastrophic illness in Taiwan. The patients were followed up until the diagnosis of RA, withdrawal from the NHI program, or the end of 2013. The comorbidities analyzed in this study were hypertension (ICD-9-CM codes 401–405), diabetes mellitus (ICD-9-CM code 250), hyperlipidemia (ICD-9-CM code 272), coronary artery disease (CAD, ICD-9-CM codes 410–414), chronic obstructive pulmonary disease (COPD, ICD-9-CM codes 491, 492, 496), asthma (ICD-9-CM code 493), cancer (ICD-9-CM codes 140–208), allergic rhinitis (ICD-9-CM codes 477, 472.0), atopic dermatitis (ICD-9-CM code 691), chronic liver diseases (ICD-9-CM code 571.4), hepatitis B (ICD-9-CM codes 070.2, 070.3, V02.61), and hepatitis C (ICD-9-CM codes 070.41, 070.44, 070.51, 070.54, V02.62).

### Statistical analysis

The chi-squared test was used for category variables and student’s t-test for continuous variables. The number of person-years was calculated from the sum of the follow-up time for each individual and the follow-up time was defined as the period from the index date to the diagnosis of RA, withdrawal from the NHI program, or the end of 2013. The incidence rate was calculated according to the number of occurrences and person-years. Hazard ratios (HRs) and 95% confidence intervals (CIs) of the two groups were estimated using univariate and multivariate Cox proportional hazard regression models. The variables in the multivariate model included age, gender and all comorbidities. The Kaplan-Meier method was used to describe the cumulative incidence of RA among the two groups; differences between the two groups were evaluated using the log rank test. SAS statistical software (version 9.4 for Windows; SAS Institute Inc., Cary, NC, USA) was used for data analysis, and a p-value less than 0.05 was considered to indicate statistical significance.

## Results

### Demographic characteristics and comorbidities of the patients with Mycoplasma pneumonia and the comparison cohort

The eligible participants in this study included 116,053 patients in the MP group and 464,212 patients in the comparison group. There were no significant differences in age and gender (Table 1), though there were slightly more females (52%) than males and 85.5% of the participants were under 19 years old. As shown in Table 1, the MP group had a higher incidence rate of comorbidities. The mean follow-up time in the MP group and the comparison group were 5.19 and 5.21 years respectively.

**Table 1:** Baseline characteristics of patients.

	Mycoplasma pneumonia				p-value*
	Yes		No		
	(n = 116053)		(n = 464212)		
	n	%	n	%	
**Gender**					>0.99
Male	557374	48.0	222936	48.0	
Female	60319	52.0	241276	52.0	
**Age**					>0.99
≦19	99251	85.5	397004	85.5	
20–39	7056	6.08	28224	6.08	
40–64	4914	4.23	19656	4.23	
≧65	4832	4.16	19328	4.16	
mean(SD)	12.0 (18.0)		12.0 (18.0)		0.93^a^
**Comorbidity**					
Hypertension	4824	4.16	8307	1.79	<0.0001
Diabetes mellitus	2819	2.43	4145	0.89	<0.0001
Hyperlipidemia	1428	1.23	1994	0.43	<0.0001
CAD	2298	1.98	3725	0.80	<0.0001
Hepatitis B	625	0.54	522	0.11	<0.0001
Hepatitis C	479	0.41	401	0.09	<0.0001
Cancer	854	0.74	2377	0.51	<0.0001
Allergic rhinitis	7426	6.40	3285	0.71	<0.0001
Chronic liver diseases	437	0.38	476	0.10	<0.0001
Atopic dermatitis	7470	6.44	9543	2.06	<0.0001
Asthma	19378	16.7	10234	2.20	<0.0001
COPD	2993	2.58	2134	0.46	<0.0001

CAD, coronary artery disease; COPD, chronic obstructive pulmonary disease

*Chi-square test

^a^ t-test

### Comparison of the incidence and HR of RA stratified by gender and age of the patients with Mycoplasma pneumonia and the comparison cohort

**Fig 1:**
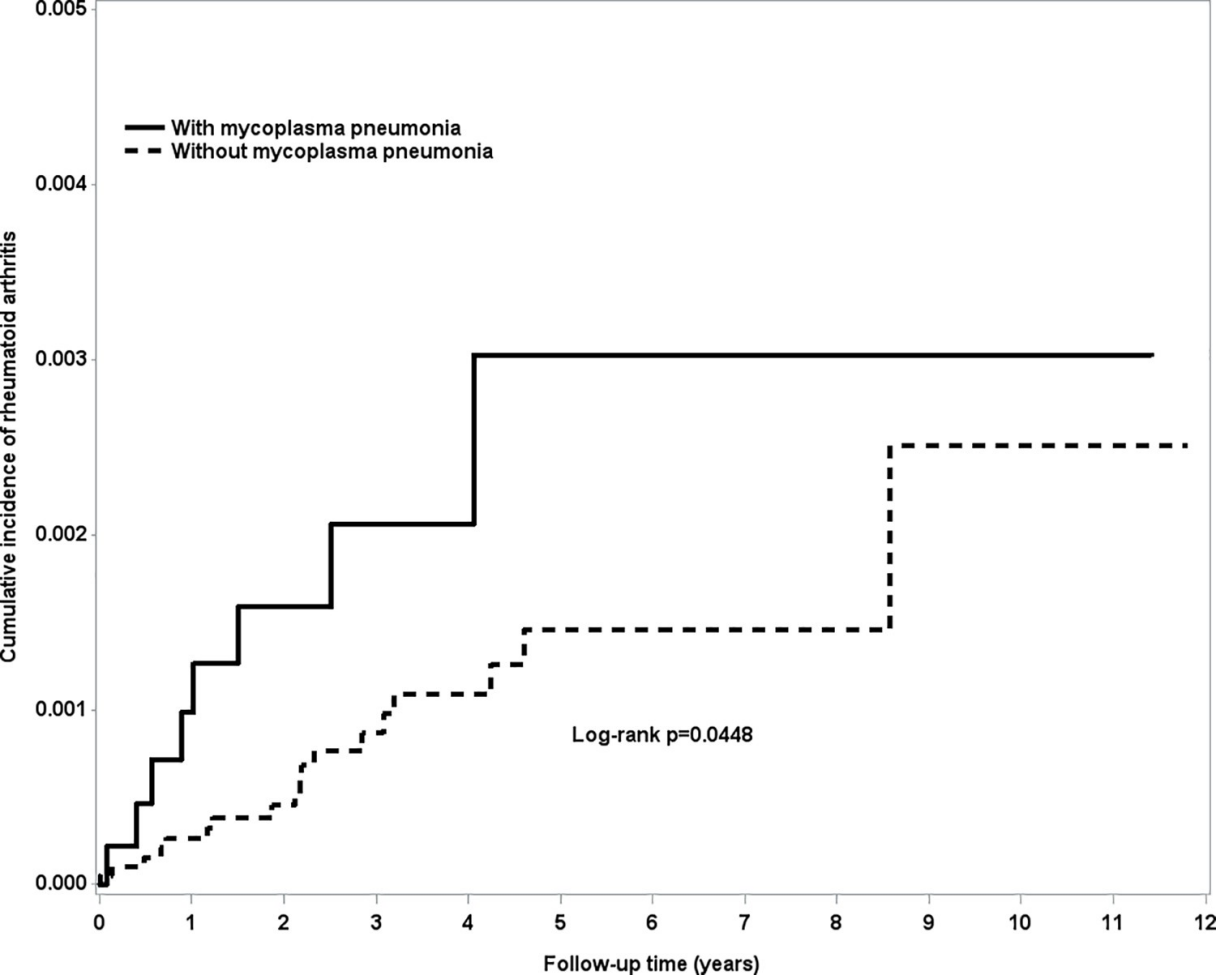
The cumulative incidence of rheumatoid arthritis in elderly patients with and without mycoplasma pneumonia.

The incidence rates of RA in the MP group and the control group were 4.98 and 3.68 per 100,000-person year respectively. The adjusted HR of RA for the MP group was 1.37 (95% CI = 0.87–2.16) in comparison to the control group (Table 2). Compared to the female subjects without MP, those with MP had a non-significant 1.61-fold higher risk of RA (95% CI = 0.92 to 2.83), as shown in Table 2. In the group over 65 years old, the adjusted HR for the MP group was 3.05 (95% CI 1.16–7.99, p = 0.02). The survival curve (Fig 1) showed that the cumulative incidence of RA in those over 65 was higher in the MP group than in the control group (p<0.05). The adjusted HR(95% CI) of RA for MP group in female older than 65 years was 2.38(0.71,8.05) (p = 0.16); while adjusted HR(95% CI) of RA for MP group in male older than 65 years was 3.63(0.75,17.6) (p = 0.11).For the patients without any comorbidities, the MP group had a non-significant 1.41-fold higher risk of RA (95% CI = 0.78 to 2.52), as seen in Table 2. The incidence rate of RA in the MP group without cancer, and allergic diseases such as asthma, allergic rhinitis and atopic dermatitis was 3.80 per 100,000-person year; however, the incidence rate of RA in the MP group with any of above disease was 8.37. The adjusted HR = 1.77 (95% CI 0.24–12.8, p = 0.57); there is no significantly statistical difference between these 2 groups.

**Table 2:** Incidence and hazard ratio of rheumatoid arthritis in patients with mycoplasma pneumonia compared to controls, stratified by gender, age, and comorbidity.

	Mycoplasma pneumonia						Compared to Control			
	Yes			No			Crude		Adjusted	
	event	PY	IR	event	PY	IR	HR (95% CI)	p-value	HR (95% CI)	p-value
**Overall**	30	602339	4.98	89	2419324	3.68	1.35 (0.89, 2.04)	0.16	1.37 (0.87, 2.16)	0.18
**Gender**										
Male	9	287656	3.13	37	1161586	3.19	0.98 (0.47, 2.04)	0.96	0.98 (0.44, 2.16)	0.95
Female	21	314683	6.67	52	1257737	4.13	1.61 (0.97, 2.67)	0.07	1.61 (0.92, 2.83)	0.09
**Age**										
≦19	13	535702	2.43	37	2130327	1.74	1.39 (0.74, 2.61)	0.31	1.20 (0.61, 2.36)	0.60
20–39	3	33931	8.84	9	135268	6.65	1.33 (0.36, 4.93)	0.66	0.85 (0.18, 3.95)	0.84
40–64	6	19871	30.19	25	85596	29.21	1.04 (0.43, 2.54)	0.93	0.70 (0.24, 2.07)	0.52
≧65	8	12835	62.33	18	68132	26.42	2.29 (1.00, 5.28)	0.05	**3.05 (1.16, 7.99)**	**0.02**
**Comorbidity**^**§**^										
No	14	428638	3.27	69	2267588	3.04	1.07 (0.60, 1.90)	0.82	1.41 (0.78, 2.52)	0.25
Yes	16	173701	9.21	20	151736	13.2	0.69 (0.36, 1.34)	0.27	1.08 (0.52, 2.27)	0.83

PY: person-years; IR: incidence rate, per 100,000-person years; HR: hazard ratio; CI: confidence interval

Model was adjusted by gender, age, and all comorbidities listed in Table 1.

Comorbidity^§^: Patients with any one of the comorbidities were classified as the comorbidity group

### Risk of RA according to follow-up years by subgroup of age

Table 3 shows the incidence and HRs of RA stratified by follow-up years and subgroup of age. Among all age subgroup, it was shown that higher risk of subsequent development of rheumatoid arthritis among 2 age groups of MP victims in the first two years. A 4.14-fold significant increase in the risk of developing RA was observed within the first 2 years of follow-up (95% CI = 1.27,13.4, p value 0.02) among the MP group aged 65 and older. The adjusted HR of RA for the MP group aged 19 and younger was 3.19 (95% CI = 1.04.9.76, p value 0.02) within the first 2 years of follow-up.

**Table 3:** Incidence and hazard ratio of rheumatoid arthritis stratified by follow-up year.

	Mycoplasma pneumonia								Compared to Control			
	Yes				No				Crude		Adjusted	
Follow time	N	event	PY	IR	N	event	PY	IR	HR (95%CI)	p-value	HR (95%CI)	p-value
**Patients aged≦19 years**												
** <2**	99251	5	193644	2.58	397004	8	770902	1.04	2.49(0.81,7.61)	0.11	3.19(1.04,9.76)	0.04
** 2–5**	88322	5	208039	2.40	352574	17	827436	2.05	1.17(0.43,3.17)	0.76	0.77(0.25,2.31)	0.64
** ≧5**	50314	3	134019	2.24	200798	12	531990	2.26	0.97(0.27,3.44)	0.96	0.80(0.21,3.07)	0.75
**Patients aged 20–39 years**												
** <2**	7056	1	13459	7.43	28224	2	53763	3.72	2.00(0.18,22.1)	0.57	2.56(0.23,28.3)	0.44
** 2–5**	5895	2	13070	15.3	23637	4	52012	7.69	1.99(0.36,10.9)	0.43	0.77(0.09,6.52)	0.81
** ≧5**	2886	0	7402	0.00	11667	3	29493	10.2	-		-	
**Patients aged 40–64 years**												
** <2**	4914	2	8947	22.4	19656	8	37303	21.4	1.04(0.22,4.92)	0.96	0.93(0.16,5.52)	0.94
** 2–5**	3699	3	7454	40.2	16012	10	32626	30.7	1.31(0.36,4.76)	0.68	0.55(0.12,2.66)	0.46
** ≧5**	1466	1	3469	28.8	6634	7	15667	44.7	0.64(0.08,5.21)	0.68	0.46(0.03,6.39)	0.56
**Patients aged ≥ 65 years**												
** <2**	4832	6	7144	84.0	19328	8	34525	23.2	3.55(1.23,10.2)	0.02	4.14(1.27,13.4)	0.02
** 2–5**	2516	2	4288	46.6	13609	9	24524	36.7	1.26(0.27,5.85)	0.09	1.47(0.22,10.1)	0.69
** ≧5**	655	0	1402	0.00	4088	1	9083	11.0	-		-	

PY: person-years; IR: incidence rate, per 100,000-person years; HR: hazard ratio; CI: confidence interval

Model was adjusted by gender, age, and all comorbidities listed in Table 1.

## Discussion

This study was the first retrospective cohort study using nationwide population-based data to evaluate the risk of developing RA associated with MP infection. The results demonstrated a 3.1-fold higher risk of RA (95% CI = 1.16–7.99) among the MP group aged 65 and older. The patients diagnosed with MP were also observed to have a prominent risk of developing RA in the first 2 years, compared with the general population, especially in 2 age groups (≦19 years and ≥ 65 years). Hence, the high-risk groups of MP patients should be appropriately monitored. Furthermore, the effects of MP were found to be more prominent, though not statistically significant, in the female patients (adjusted HR 1.61, 95% CI = 0.92 to 2.83). Although the patients with MP had a significantly higher rate of comorbid diseases compared to the comparison cohort, MP remained an independent risk factor for developing RA among the elderly, following adjustment for gender and comorbidities.

This study is noteworthy for several reasons. Although multiple factors are thought to contribute to the development of RA, immunological studies on animal models of RA have strongly suggested that infections represent the main environmental triggers for human RA [16, 25]. Relevant population-based epidemiological study data to support this claim is not yet available. This study involved a cohort study design with minimal risk of recall bias and had a longer follow-up period.

A recently published population-based case-control study (involving 6,401 participants with 2,831 cases and 3,570 controls) in Sweden reported no increased risk of RA with antecedent infections [25] and that antecedent urinary tract infections and gastroenteritis were associated with a reduced risk of incident RA [25]. No persuasive explanation for these findings was given, but interestingly, the findings indicated that infections by gram-negative bacteria contributed to a decrease in risk of RA while infections by gram-positive bacteria did not[25]. With a case control design and an exposure duration recorded by self-reported questionnaire, the study could not make definitive conclusions due to the recall bias and uncontrolled confounding factors.

This study found the most elderly group with MP, which often developed during their middle-age, had an increased risk of RA. But the prominent risk of developing RA was only observed in the first 2 years. This may be due to the duration of T-cell memory effect, which is dependent on the patients’ pathogen immunogenicity, host immunity, and age, suggesting that the elderly patients had a shorter T-cell memory.

The underlying mechanism by which MP increases the risk of developing RA among the elderly is unclear. As demonstrated in some animal models, mycoplasma species can cause acute or chronic arthritis in rodents via one of the “superantigens,” which activate T cells to express many different beta genes [26–28]. Mycoplasmas exhibit many effects on the immune cells and immune system of the host, including polyclonal activation of T and B cells as well as the associated secretion of cytokines [26]. However, it remains uncertain whether the immunopathological process is elicited through molecular mimicry or modulation of the immune response, in which both immune cell activation and cytokine production cause a cytokine imbalance [10, 29]. Recent studies have revealed that mycoplasma causes cytokine reaction with Th2 and Th17, including IL1, IL6, and IL17, alongside an imbalance in regulatory T cells [30, 31].

Several limitations regarding the analysis of the results have to be stated. First, the ICD-9-CM codes for the diagnosis of MP were based on administrative claims data recorded by physicians and hospitals instead of a prospective clinical setting. Consequently, there may be some inaccuracy that could have resulted in misclassification, although the Bureau of NHI used an auditing mechanism to minimize diagnostic uncertainty and misclassification[24]. In addition, the diagnosis of RA in this study was strictly defined using the catastrophic illness database in order to yield better diagnostic validity. Second, some potential confounding factors of RA like obesity, smoking, socioeconomic status, and geographic differences were not covered in this study, except for COPD, which has been used in several previous studies [32, 33] as a proxy variable for cigarette smoking. Third, other potential confounding factors including inflammatory biomarkers and family history were not taken into consideration in this study. Finally, it remains uncertain whether the findings in this study can be generalized to other ethnic groups, as the majority of the participants were Taiwanese.

## Conclusion

This 13-year population-based cohort study demonstrated a higher risk of RA in patients with MP, especially among patients aged older than 65.This higher risk were noted among 2 age groups of MP victims (≦19 years and ≥ 65 years) in the first two years following diagnosis of MP. Future studies are required to clarify the underlying biological mechanisms of these associations. We suggest that clinicians should provide appropriate monitoring of RA in patients with MP.
